# Rapid and Sensitive Detection of Shiga Toxin-Producing *Escherichia coli* (STEC) from Food Matrices Using the CANARY Biosensor Assay

**DOI:** 10.3390/toxins16070325

**Published:** 2024-07-22

**Authors:** Christina C. Tam, Wen-Xian Du, Yangyang Wang, Andrew R. Flannery, Xiaohua He

**Affiliations:** 1 Foodborne Toxin Detection and Prevention Research Unit, Western Regional Research Center, Agricultural Research Services, United States Department of Agriculture, 800 Buchanan Street, Albany, CA 94710, USA; christina.tam@usda.gov (C.C.T.); wen-xian.du@usda.gov (W.-X.D.); 2 Smiths Detection, 2202 Lakeside Blvd, Edgewood, MD 21040, USA; yangyang.wang@smiths-detection.com (Y.W.); andrew.flannery@smiths-detection.com (A.R.F.)

**Keywords:** Shiga toxins, STEC, biosensor, CANARY^®^ detection, B-cell based assay, immunoassay, food safety

## Abstract

Shiga toxin-producing *Escherichia coli* (STEC) causes a wide spectrum of diseases including hemorrhagic colitis and hemolytic uremic syndrome (HUS). Previously, we developed a rapid, sensitive, and potentially portable assay that identified STEC by detecting Shiga toxin (Stx) using a B-cell based biosensor platform. We applied this assay to detect Stx2 present in food samples that have been implicated in previous STEC foodborne outbreaks (milk, lettuce, and beef). The STEC enrichment medium, modified Tryptone Soy Broth (mTSB), inhibited the biosensor assay, but dilution with the assay buffer relieved this effect. Results with Stx2a toxoid-spiked food samples indicated an estimated limit of detection (LOD) of ≈4 ng/mL. When this assay was applied to food samples inoculated with STEC, it was able to detect 0.4 CFU/g or 0.4 CFU/mL of STEC at 16 h post incubation (hpi) in an enrichment medium containing mitomycin C. Importantly, this assay was even able to detect STEC strains that were high expressors of Stx2 at 8 hpi. These results indicate that the STEC CANARY biosensor assay is a rapid and sensitive assay applicable for detection of STEC contamination in food with minimal sample processing that can complement the current Food Safety Inspection Service (US) methodologies for STEC.

## 1. Introduction

*Shigella dysenteriae* type I produces the critical virulence factor Shiga toxin (Stx) that can cause mammalian cell cytotoxicity, and some pathogenic *Escherichia coli* (*E. coli*) strains have been found to harbor one or more of these Shiga-like toxins. Data collected by the Centers for Disease Control and Prevention (US) between 2009–2021 show that there have been 1019 Shiga toxin-producing *Escherichia coli* (STEC) outbreaks leading to 14,010 illnesses, 2218 hospitalizations, and 43 deaths [1]. STEC infections can progress in severe cases to hemorrhagic colitis (HC) and hemolytic uremic syndrome (HUS) due to the activity of the Stxs produced by these strains [2,3,4]. Stxs are AB toxins consisting of a single catalytic A-subunit and five B-subunits important for receptor binding. Stxs are members of the type II family of ribosome inhibiting proteins (RIP) and enzymatically inactivate the eukaryotic ribosome to inhibit protein synthesis. These toxins are categorized into two types: Stx1 (subtypes: Stx1a, Stx1c, Stx1d, and Stx1e) and Stx2 (Stx2a–i, and Stx2k) [5,6,7,8,9,10]. Though four Stx subtypes (Stx1a, Stx2a, Stx2c, Stx2d) correlate highly with human disease, other Stx subtypes have also been implicated [11,12,13,14,15]. The thermal stability of Stxs is of major concern since normal food pasteurization conditions may kill STEC bacteria but leave biologically active Stxs present in foods to be consumed [16,17,18,19,20,21]. Early, rapid, and sensitive detection of STEC/Stx in food and environmental samples will greatly reduce illness, hospitalization, and potentially death due to the lack of FDA-approved therapeutics.

There is an extensive list of technologies that are used to detect Stxs including PCR, ELISA, LC-MS, mouse bioassays, cell-free assays, cell-based assays, biosensors, etc. [22,23,24,25,26,27,28,29,30,31,32,33,34,35,36,37,38,39,40,41]. All of these detection strategies have their own advantages and disadvantages, thus having multiple detection platforms available for use is critical. Multiple PCR-based assays including real-time PCR have been developed to detect the presence of *stx* genes to indicate contamination with STEC. However, these PCR-based assays detect the presence of the toxin genes but not the toxin itself, and depending on the primers used, the assay may not detect all Stx subtypes due to the genetic polymorphisms of the *stx* genes. The sensitivity of the PCR-based assays at best ranges from 1–10 CFU/g/reaction which is higher than our targeted biosensor assay range [26,27,28,30,33,36,37]. In a previous study, our laboratory determined a limit of detection (LOD) of 290 ng of Stx2 per kilogram of mouse body weight using the in vivo mouse bioassay which can model all aspects of Stx intoxication including antibody protection [41]. However, the in vivo mouse bioassay is laborious, time-consuming, requires d for read-out and is expensive. Immunoassays such as ELISAs have detected Stx with high sensitivity (LOD ≈ 25 pg/mL) in a shorter period (2.5 h as compared to d for the mouse bioassay). Mass spectrometry is a powerful tool, and an LC-MS method was able to detect 5 fmol/mL, but it does not distinguish between active or inactive Stx and has a high instrument cost requiring specialized training to operate. In vitro cell-free translation assays have been developed to detect biologically active Stx with high sensitivity and reproducibility but are not able to model all aspects of Stx intoxication [25]. Cell-based assays are another detection platform and a cell-based cytotoxicity assay detected STEC contamination at 0.4 CFU/mL or less in drinking and environmental water sources, but the read-out was two d, thus impairing early diagnosis and treatment [22]. 

Currently, the United States Department of Agriculture, Food Safety, and Inspection Service (FSIS) uses the Food and Drug Administration (FDA) Bacteriological Analytical Manual (BAM) method, Chapter 4 [42] for the screening and detection of diarrheagenic *E. coli*. This method involves sample preparation of various foods followed by enrichment using specific media for 18–24 h. The overnight enrichment samples can be subjected to real-time PCR and/or further culture confirmation for STEC involving cell plating on differential agar to confirm the STEC isolates. This process is repeated with the putative STEC isolate recovered from the overnight enrichment media increasing the time needed for diagnosis and delaying initiation of product recall and/or confirmation of STEC infection and treatment. Due to the complexity involved in STEC outbreaks, multiple technologies may be required for early diagnosis and treatment of STEC infection.

Recently in our laboratory, we have developed a STEC CANARY^®^ (Cellular Analysis and Notification of Antigen Risks and Yields) cell-based biosensor assay for the detection of Shiga toxin 2 [43]. The STEC biosensor assay was rapid (≈3 min), highly sensitive (LOD of ≈0.1–0.2 ng/mL), reproducible, recognized most Stx2 subtypes with no cross-reactivity to Stx1a, ricin, or abrin, and had a long shelf-life of up to 10 d after reconstitution with minimal loss in detection capability [43]. Additionally, the STEC biosensors were able to detect Stx2 from STEC culture supernatants at 8– and 24–h post-inoculation under optimal laboratory conditions. In this study, we wanted to evaluate the feasibility and effectiveness of the CANARY^®^ B-cell-based biosensor assay for the detection of Stx2 from STEC-contaminated foods.

## 2. Results

### 2.1. Schematic of STEC Biosensor Assay for Milk, Beef, and Lettuce

**Figure 1:**
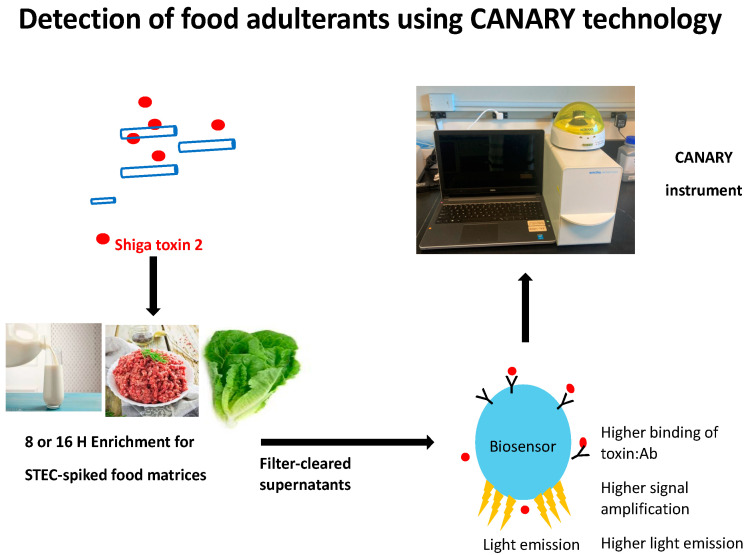
STEC CANARY^®^ biosensor assay workflow for food matrices. The assay detects the presence of Shiga toxin 2 expressed from STEC bacteria spiked into relevant known foodborne outbreak matrices such as milk, beef, and lettuce. For STEC and negative control bacteria food samples, enrichment in mTSB with mitomycin C for either 8– or 16–h was initiated. Filtered cleared supernatants from either Stx2 toxoid-spiked or bacteria-spiked samples were added to STEC biosensors. STEC B-cell biosensors express Stx2-specific antibodies on their cell surface and bind to the antigen of interest, thus initiating a signal transduction cascade triggering light emission. As more antigen is bound to the cell surface displayed antibody receptors, amplification of the signal is induced, and more light is emitted. This light emission is detected using the CANARY instrument.

Figure 1 depicts a workflow of the STEC CANARY^®^ biosensor assay for food matrices. Biosensors expressing membrane-bound antibodies that are specific to Stx2 were assessed for their ability to bind to and detect Stx2 toxoid and Stx2 toxin produced natively by STEC strains. Filter-cleared supernatants from either Stx2 toxoid spiked food samples or STEC enrichment samples were added with STEC biosensors, and the assay was initiated. The luminometer detects the light output, which is expressed as relative light units (RLU) over time (120 s, read every second). The binding of Stx2 to multiple surface-expressed antibodies initiates an amplified signal transduction event leading to differential light expression levels. 

### 2.2. STEC Biosensors Sensitively Detect Stx2 Toxoid in mTSB after Sample Dilution

**Figure 2:**
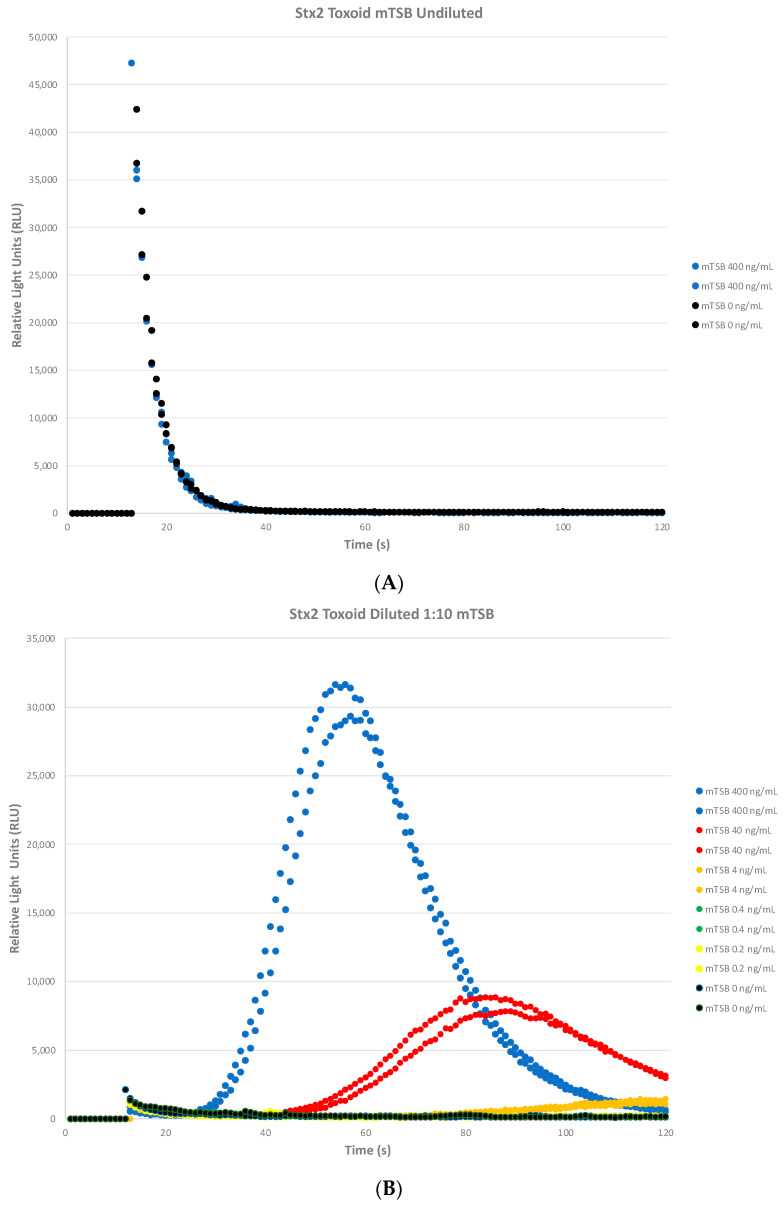

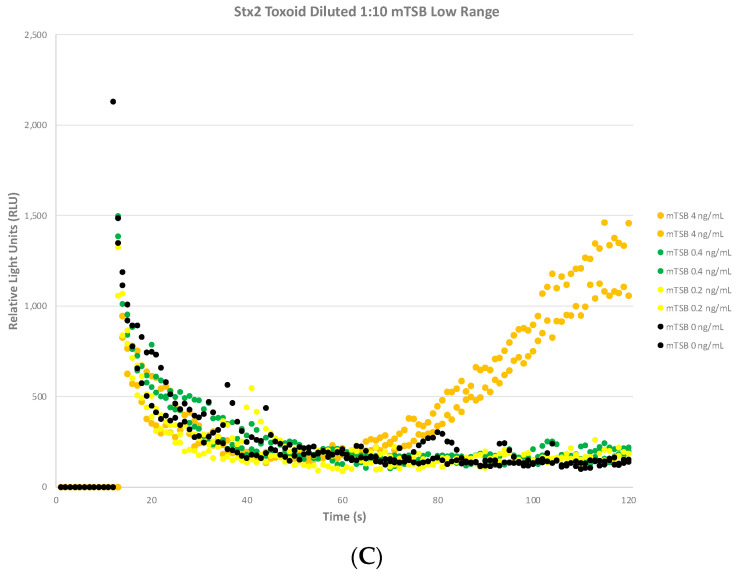
Growth media interference with the biosensor assay. (**A**) Sample buffer consisting of modified TSB (mTSB) spiked with Stx2a toxoid inhibited the STEC biosensor assay (blue circles 400 ng/mL compared to mTSB 0 ng/mL black circles). (**B**) A 1:10 dilution with assay buffer was required to abrogate this matrix effect. A rapid and sharp increase and then decrease of the highest Stx2a toxoid concentration is seen (400 ng/mL, blue circles). As the concentration decreases, the curves shift right along with a decrease in relative light units (RLU). (**C**) The lower concentrations of Stx2a toxoid are shown with the estimated limit of detection (LOD) at ≈4 ng/mL (gold circles). The RLU starts increasing approximately 70 s onwards and may plateau around 120 s with an RLU above 600 RLU (3* RLU of negative 0 ng/mL mTSB, black circles). Each independent assay consisted of duplicate samples per dose with two independent experiments performed.

Our previous study [43] revealed that a 1:10 dilution in the assay buffer was required for the STEC biosensor assay to detect natively expressed Stx2 from STEC overnight cultures in buffered peptone water (BPW). Since we will be growing these *E. coli* cultures in modified tryptone soy broth (mTSB) for enrichment, we wanted to determine if a matrix effect was seen with the media. Samples consisting of modified TSB (mTSB) spiked with 400 ng/mL of Stx2a toxoid (blue circles) did not show an RLU increase as expected and were flat similar to the 0 ng/mL mTSB negative control (black circles) depicted in Figure 2A. Figure 2B shows that a ten-fold dilution with assay buffer abrogated this matrix effect and restored the responsiveness of the biosensor assay. A rapid and sharp increase and then decrease at the highest Stx2a toxoid concentrations is seen (400 ng/mL, blue circles). As the concentration decreases, the curves shift right along with a decrease in relative light units (RLU). As the Stx2a concentrations decrease as depicted in Figure 2C, the RLU starts increasing approximately 70 s onwards (4 ng/mL, gold circles) and may plateau around 120 s. We estimate the LOD at ≈4 ng/mL (gold circles) with an RLU above 600 RLU (3* RLU of negative 0 ng/mL mTSB, black circles).

### 2.3. Detection of Stx2a in Toxoid-Spiked Food Matrices

Since we were able to establish that the biosensor assay was able to detect 4 ng/mL of Stx2a toxoid in mTSB as seen in Figure 2B,C, we wanted to determine the assay’s ability to detect Stx2a toxoid spiked in food matrices. Beef (98% lean), milk (2% fat), and romaine lettuce are foods that have been known to be involved in STEC outbreaks. From our previous study that determined the sensitivity of the assay under optimal laboratory conditions, Stx2a toxoid at various concentrations (400 ng/mL, 40 ng/mL, 4 ng/mL, 0.4 ng/mL, 0.2 ng/mL, and 0 ng/mL) was spiked into these three food matrices. For all the food matrices, the biosensor assay was able to detect Stx2a toxoid reproducibly and sensitively to 4 ng/mL (Table 1) with RLU curves similar to those shown in Figure 2B,C.

### 2.4. STEC Biosensors Rapidly and Sensitively Detect Natively Expressed Stx2 in STEC-Contaminated Foods after Enrichment

#### 2.4.1. Detection of Stx2 from STEC-Contaminated Milk and Beef after Enrichment for 8– or –16 h

Since we have shown in this study that the biosensor assay could detect Stx2a toxoid spiked into relevant food matrices, the key test would be to assess its detection capability for Stx2 expressed natively by STEC strains in contaminated food after enrichment. Using the FDA BAM Chapter 4 methodology used by FSIS, all samples (beef, milk, and romaine lettuce) were prepared similarly. *E. coli* strains ATCC25922 (O6, *stx-*), RM5856 (O121:H19), and RM9872 (O145) derived from single colonies were grown overnight before dilution and inoculated in enrichment media with 100 ng/mL mitomycin C into all three food matrices with a target ≈0.4 CFU/g or 0.4 CFU/mL. The actual inoculum determined by cell plating is shown in Table 2. The STEC-inoculated food samples were stomached and enriched for 8– or –16 h. The centrifuged and filtered enrichment samples were subjected to a biosensor assay.

**Figure 3:**
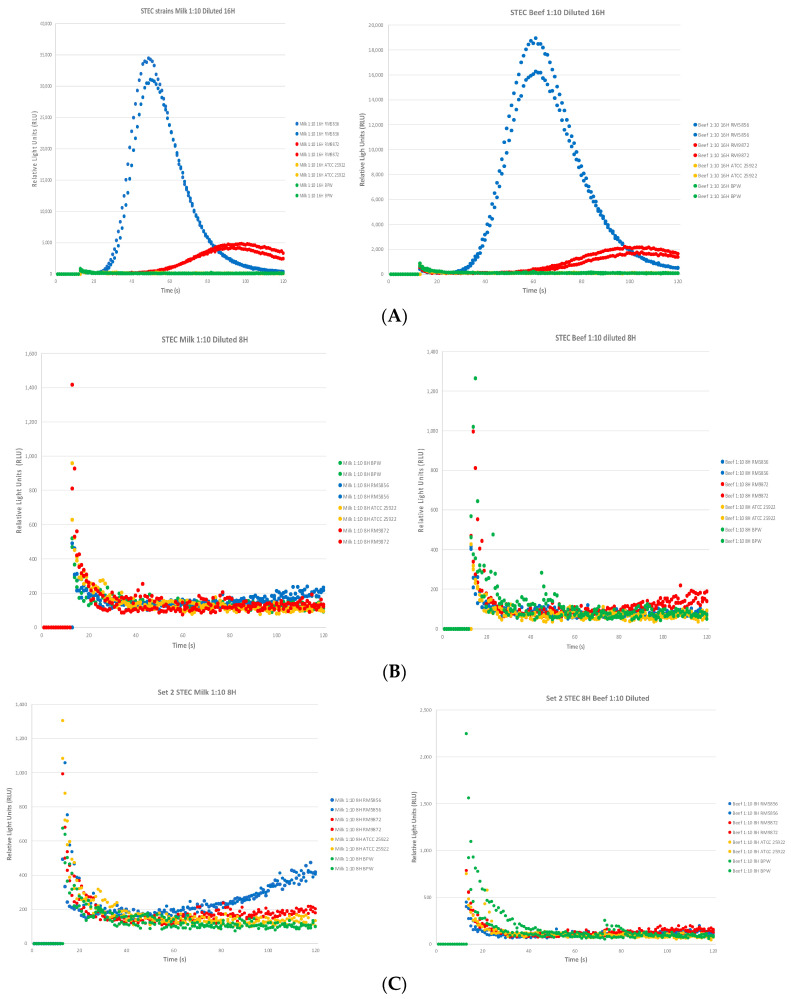
Detection of Stx2 expressed from STEC strains in contaminated milk and beef after enrichment. (**A**) STEC strains RM5856 (high expressor), RM9872 (low expressor) were spiked into beef and milk samples at a concentration of 0.4 CFU/g or 0.4 CFU/mL. The biosensor assay was able to detect Stx2 expressed by RM5856 (blue circles) and RM9872 (red circles) after 16 h of enrichment with RM5856 having the highest Stx2 expression and hence RLU curve in experiment 1. (**B**) In the 8 h enrichment samples from experiment 1, relatively flat curves were observed for all strains. (**C**) For experiment 2, a slight increase in the RM5856 (blue circles) is seen from 60 s onwards but still below our estimated cut-off of 600 RLU in the milk sample (left graph), but no such increase is observed for the beef (right graph). The 16 h results from experiment 2 were similar in trend from experiment 1. Each independent assay consisted of duplicate samples per dose with two independent experiments performed.

The biosensor assay was able to detect Stx2 from both RM5856 (blue circles) and RM9872 (red circles) as compared to the ATCC25922 negative control strain (green circles) in the beef and milk samples after 16 h enrichment. As expected, RM5856 expressed more Stx2 than RM9872 (Figure 3A), corroborating our results from the previous study using bacterial overnight cultures. For the 8 h results, there were variabilities seen between two independent experiments shown in Figure 3B,C. In Figure 3B, the assay shows flat RLU curves for all strains in milk and beef samples. For Figure 3C, depicting a separate independent experiment performed on a different day, we can see an increase starting at about 60 s for the RM5856 (high expressing strain, blue circles) compared to RM9872 (red circles) or ATCC25922 negative control (green circles). Interestingly, we see the variability specifically for RM5856, a known high Stx2 expressing strain, in the milk but not beef samples in the set 2 experiments. In the set 2 experiments, the inoculum for all three strains was similar ranging from 0.14 cfu/g/mL–0.19 cfu/g/mL, suggesting the differences were not due to differential CFUs spiked into the food matrices that would account for the results. In the set 1 experiments, the RM5856 inoculum was 0.38 CFU/g/mL, which is two-fold higher than in set 2. The results from Figure 3C would suggest potentially that: (1) a lower seeding density may be desirable for optimal Stx2 expression for RM5856 early or (2) RM5856 in set 1 was expressing sufficient amounts of Stx2 but the milk matrix may have inhibited the toxin from interacting with the biosensors due to the slight trending increase in the RLU curve for RM5856 in Figure 3B from 80 s onwards. However, we have to still consider the 8 h sample to be negative since our estimated cut-off for positivity is about 600 RLU. The 16 h enrichment samples for the experiment held a similar trend to those seen in experiment 1 and were not depicted.

#### 2.4.2. Detection of Stx2 from STEC-Inoculated Lettuce after 8– or –16 h Enrichment

**Figure 4:**
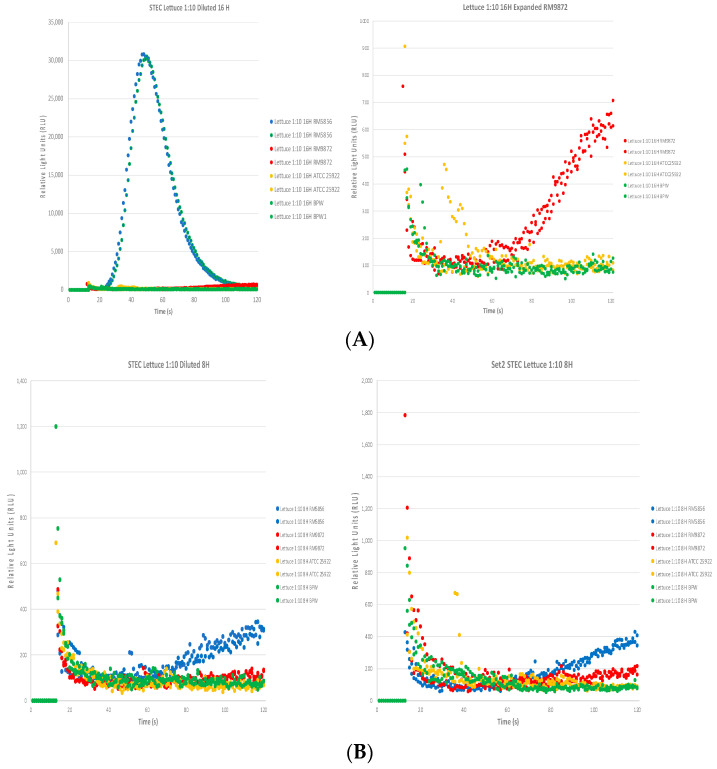
Detection of Stx2 expressed from STEC strains in romaine lettuce after enrichment. (**A**) STEC strains RM5856 (high expressor) and RM9872 (low expressor) were spiked into romaine lettuce samples at a concentration of 0.4 CFU/g or 0.4 CFU/mL. The biosensor assay was able to detect Stx2 expressed by RM5856 (blue circles, left graph) and RM9872 (red circles, expanded curve right graph) after 16 h of enrichment, with RM5856 having the highest Stx2 expression, hence the RLU curve in experiment 1. (**B**) In the 8 h enrichment samples from experiment 1, a gradual increase in the RLU curve for RM5856 (blue circles, left graph) was observed below our current estimated cut-off of 600 RLU (3* above RLU of negative control, ATCC25922 yellow circles). For experiment 2, as depicted in (**B**) (right graph), a similar trend was observed for RM5856 (blue circles) with the RM9872 curve potentially trending slightly above from 100 s onwards but currently estimated to be negative. The 16 h results from experiment 2 were similar in trend to experiment 1. Each independent assay consisted of duplicate samples per dose with two independent experiments performed.

Figure 4A shows that the STEC biosensor assay was able to detect Stx2 expressed by RM5856 (blue circles, left graph) and RM9872 (red circles, expanded curve right graph) after 16 h of enrichment in romaine lettuce, with RM5856 having the highest Stx2 expression, hence the RLU curve in experiment 1. For the 8 h enrichment samples from experiment 1 shown depicted in Figure 4B, a gradual increase in the RLU curve for RM5856 (blue circles, left graph) was observed but below our current estimated cut-off of 600 RLU (3* above RLU of negative control, ATCC25922 yellow circles). For experiment 2 as depicted in Figure 4B (right graph), a similar trend was observed for RM5856 (blue circles) with potentially the RM9872 curve trending slightly above from 100 s onwards but too low to be considered positive. The results for the RM5856 8 h samples from the romaine lettuce show in both experiments that the RLU curves are increasing, though conservatively considered negative, suggesting that the food matrix is most likely responsible for the variable results. It suggests since RM5856 expresses a higher level of Stx2 than RM9872 even at 8 h, the Stx2 toxin may be sequestered by the food itself or a component of it (milk fat, fat from beef, romaine lettuce) and hence is not able to bind in sufficient quantities to give an enhanced signal. The 16 h results from experiment 2 were similar in trend to experiment 1. The results for the detection of Stx from STEC-contaminated food samples after enrichment are summarized in Table 3.

## 3. Discussion

Detection of Shiga toxins utilizes multiple methods including antibody-based, mass-spectrometry, cell-based assays, cell-free assays, as well as in vivo mouse assays. Depending on the sensitivity needed and detection in complex matrices such as food and/or environmental samples, multiple detection methodologies may be required. Recently, we characterized a STEC biosensor assay that was rapid (3 min), reproducible, and sensitive with limits of detection ≈0.1–0.2 ng/mL for Stx2a toxoid, and Stx2 from bacterial culture supernatants under optimal laboratory conditions [43]. However, the utility of the biosensor assay in the detection of STEC/Stx in food or environmental sources was not determined. 

In this study, we evaluated the sensitivity and applicability of the STEC biosensors to detect Stx2 from food samples mimicking real-world applications. This is the first study using the CANARY^®^ biosensor system to detect Shiga toxins in food matrices such as beef, milk, and romaine lettuce with good sensitivity (LOD ≈ 4 ng/mL), and identified food contamination with STEC within 16 h of enrichment and potentially as early as 8 h depending on the STEC strain present and their Stx expression levels. The advantages of this assay are: (1) good sensitivity, (2) ease of use, (3) rapidity (3 min to read-out), (4) minimal sample preparation, (5) small volumes required for the assay (≈200 μL), (6) compatibility with complex food matrices, and (7) lower or comparable instrument costs compared to other methodologies.

Similar to other detection methods, the STEC biosensor assay also has some disadvantages. One disadvantage of the assay in its current format is the inability to multiplex different toxins or distinguish which Stx2 variant is detected due to the same output via light emission as a read-out compared to other assays such as PCR/qRT-PCR, multi-toxin lateral flow immunoassays, or Luminex multiplex detection assays. Another assay disadvantage would lie in its dependence on the level of Stx and subtype produced by *E. coli* in the samples of interest, as compared to detection based on the toxin genes via PCR/qPCR for instance. Our previous study indicated that these biosensors could detect most Stx subtypes including those closely associated with HUS (Stx2a, 2c, and 2d), but failed to detect Stx2e and Stx2f (less commonly involved in human diseases); therefore, STEC strains producing these Stx2 subtypes will be missed. As shown in this study, if the Stx2 levels were below detection levels either through differential expression (i.e., low vs. high STEC expressor as shown in Figure 3 and Figure 4, Table 3) or matrix inhibition, the assay will determine the sample to be negative thus potentially producing a false negative. Another disadvantage is that the current biosensor assay is qualitative rather than quantitative. The assay’s variabilities due to the factors discussed above reflect the real-world limitations that would require extensive assay optimization by the manufacturer to develop a proprietary algorithm that will be used to determine positive and negative samples in complex foods. Algorithm development in the future for this assay may render it semi-quantitative. Further optimization of the biosensor protocol (inclusion of magnetic-capture beads, washing of samples, generation of biosensors with a broad range of detection capability, etc.) may yield improvements in the assay’s sensitivity and applicability in complex food matrices, as seen with the 8 h sample variabilities for beef, milk, and romaine lettuce due to inherent food matrix interference. Even with these current disadvantages, this biosensor assay can be potentially incorporated and complementary to the current FSIS methodology to screen for STEC strains in food via the following: (1) test STEC in food directly without enrichment if their Stx levels are high and (2) test STEC in the enrichment samples at 16 hpi or even at 8 hpi instead of the current 18–24 hpi before plating and further screening, thus reducing the delay in STEC outbreak confirmation.

## 4. Conclusions

The STEC biosensor assay described in this study detects both Stx2 spiked in food and Stx2 expressed by STEC strains in relevant STEC outbreak foods after enrichment for 16 h with good sensitivity. The rapid assay time (≈3 min) and small volumes needed (≈200 μL) make this detection technology a potent qualitative tool in food safety surveillance programs. Biosensor assay optimization can make this assay even more sensitive and may allow for the detection of Stx2 from enrichment samples as early as 8 hpi. Additionally, this rapid and sensitive assay may be optimized for environmental surveillance of water sources, especially for irrigation or natural water reservoirs that may be contaminated by STEC strains either via run-off or through animal activity. These natural water reservoirs may be a source of irrigation water whose use may inadvertently contaminate our food supply, especially for leafy greens that have been implicated in many multistate STEC foodborne outbreaks.

## 5. Materials and Methods

### 5.1. Reagents and Preparation of Food Matrices

The Stx toxoids and holotoxins are USDA-generated materials, as described previously [29,44,45,46]. STEC biosensors and the assay buffer were produced by Smiths Detection (Smiths Detection, Edgewood, MD, USA) [43]. Toxins and enrichment supernatants were kept at −20 °C and −80 °C before use. STEC biosensors were stored in liquid nitrogen and thawed immediately before use. Ground beef (98% lean), milk (2% fat), and romaine lettuce were purchased from a local supermarket. Lettuce was chopped into ~1 cm^2^ pieces using a knife/scissor inside a biosafety hood. The CANARY^®^ detection system consists of a laptop, a small microcentrifuge (SCILOGEX D1008, Rocky Hill, CT, USA), and a luminometer (Sirius L Tube Luminometer TITERTEK-Berthold, Pforzheim, Germany).

### 5.2. Biosensor Engineering and Reconstitution of Biosensors

The heavy and light chains from Stx2 monoclonal antibodies were screened with antibiotic selection into B-cell biosensors as described in [43]. The assay buffer stored at 4 °C was removed and allowed to come to room temperature before use, as described previously. Biosensors retrieved from liquid nitrogen were immediately in a 37 °C water bath and centrifuged to remove freezing media. After centrifugation at 200× *g*, the cell pellet was reconstituted with assay buffer and counted for viable cells using trypan blue staining (T8154, Sigma, St. Louis, MO, USA). Viable biosensors were reconstituted to the defined working concentration in assay buffer, wrapped with foil, and kept at room temperature for 30 min before use. 

### 5.3. Stx2a Toxoid Detection in mTSB and Spiked Food Matrices

Toxoids were thawed at room temperature in a biological safety cabinet stored at −20 °C before use. Stx2a standards (0 ng/mL, 0.2 ng/mL, 0.4 ng/mL, 4 ng/mL, 40 ng/mL, and 400 ng/mL) were diluted with modified tryptone soy broth (mTSB, NCM0196A, Neogen, Lansing, MI, USA) to a final volume of 1.5 mL. These toxoid aliquots were added to 0.5 g/mL of each food matrix (milk, beef, and lettuce) in 15 mL tubes (50-193-1046, Fisher Scientific, San Jose, CA, USA). The samples were vortexed for 1 min and incubated on ice for 10 min before centrifugation at 10,000× *g* for 10 min. Supernatants were collected and filtered through a 0.2 μm filter (low binding SLGVR33RS, Millex, San Jose, CA, USA). Cleared supernatants were then aliquoted into 1.5 mL microcentrifuge tubes and stored at −80 °C. Supernatants were thawed at room temperature in a biological safety cabinet before use. An amount of 200 μL of each cleared and filtered food matrix supernatant was added to microcentrifuge tubes (1.5 mL 05-408-129, Fisher Scientific, San Jose, CA, USA; 2.0 mL 1420-2600, USA Scientific, Ocala, FL, USA), and then 20 μL of reconstituted room temperature STEC biosensors in foil were added to the cap of the tube. Zephyr software version 3.0 initiation is described in [43]. Duplicate samples for each food matrix and their controls were evaluated. Two independent experiments were performed. A proprietary algorithm to determine positive and negative responses based on the signal-to-noise and curve characteristics dependent on up to 28 coefficients is being developed. 

### 5.4. Biosensor Assay for the Detection of Natively Expressed Stx2 from STEC-Spiked Food Matrices

Single colonies of *E. coli* strains with or without *stx2a* [ATCC25922 O6 (*stx-*), RM5856 (O121:H19), and RM9872 (O145)] were inoculated into tryptone soy broth (TSB, Fisher Scientific, OXCM0129B, San Jose, CA, USA) and grown at 37 °C for 18 h. The overnight cultures were serially diluted to a desired CFU/mL of 10 CFU per 25 g (or mL) of samples. Actual inoculum levels were later determined by spreading 0.1 mL of cultures onto TSA (DF0369-17-6, Fisher Scientific, San Jose, CA, USA) plates and incubated overnight at 37 °C for manual colony counting. One mL of the prepared dilutions (10 cfu/mL) or BPW (R452672, Fisher Scientific, San Jose, CA, USA) control was added to each food matrix in stomacher bags (25 g or 25 mL, ~0.4 cfu/g or 0.4 cfu/mL). For the enrichment broth, 74 mL of mTSB (mTSB, NCM0196A, Neogen, Lansing, MI, USA) with 100 ng/mL of mitomycin C (BP25312, Fisher Scientific, San Jose, CA, USA) was added to the samples in stomacher bags (Seward Stomacher, 14-285-29; Classic Filter Bags 6563025, Nelson Jameson Inc., Marshfield, WI, USA) immediately before enrichment. The sample-broth solution was homogenized by a stomacher for 2 min at 230 rpm. Enrichment cultures were then incubated at 42 °C with shaking at 200 rpm for 8 and 16 h. Post enrichment, liquid cultures were removed from the stomacher bags and pipetted into 2 mL microcentrifuge tubes (1420-2600, USA Scientific, Ocala, FL, USA). After centrifugation at 10,000× *g* for 10 min at 4 °C, enrichment supernatants were collected and filtered through a 0.2 µm filter (low binding SLGVR33RS, Millex, San Jose, CA, USA). Samples were then aliquoted into 1.5 mL/tube and stored at −80 °C before testing by biosensor. The biosensor assay was performed with these enrichment samples, as described in Section 5.3. 

## Figures and Tables

**Table 1:** STEC CANARY biosensors rapidly and sensitively detected Stx2a toxoid spiked into known food outbreak matrices with a 1:10 dilution of experimental samples with assay buffer.

Stx2a Concentration	Milk	Lettuce	Beef
400 ng/mL	+	+	+
40 ng/mL	+	+	+
4 ng/mL	+	+	+
0.4 ng/mL	ND	ND	ND
0.2 ng/mL	ND	ND	ND
0 ng/mL	ND	ND	ND

ND, not detected. +, detected. Each independent assay consisted of duplicate samples per dose with two independent experiments performed.

**Table 2:** Actual inoculum of *E. coli* strains used to spike food matrices for enrichment with mitomycin C for 8– or –16 h and calculated from serially diluted agar plate counts.

*E. coli* Inoculum Stx2 Expression Levels	ATCC25922 (O6, *stx-*) None	RM5856 (O121:H19) High	RM9872 (O145) Low
Experiment 1	0.23 CFU/g/mL	0.38 CFU/g/mL	0.19 CFU/g/mL
Experiment 2	0.19 CFU/g/mL	0.19 CFU/g/mL	0.14 CFU/g/mL

**Table 3:** Detection of Stx2 using STEC biosensors from culture supernatants of milk, lettuce, and beef spiked with ≈0.4 CFU/g or 0.4 CFU/mL and grown for either 8– or –16 h.

Strains	Type	Milk 8 h	Milk 16 h	Lettuce 8 h	Lettuce 16 h	Beef 8 h	Beef 16 h
RM5856	O121:H19	- ^a^	+	- ^a^	+	- ^a^	+
RM9872	O145	-	+	-	+	-	+
ATCC25922	O6 (*stx-*)	ND	ND	ND	ND	ND	ND
Buffer Peptone Water (BPW)	Media control	ND	ND	ND	ND	ND	ND

ND, not detected. +, detected, ^a^, RLU curves increasing but currently considered negative. Each independent assay consisted of duplicate samples per dose with two independent experiments performed.

## Data Availability

The data presented in this study are available in this article.
